# Visual Fixation of Skull-Vibration-Induced Nystagmus in Patients with Peripheral Vestibulopathy

**DOI:** 10.3390/audiolres14040047

**Published:** 2024-06-24

**Authors:** Melissa Blanco, Chiara Monopoli-Roca, Marta Álvarez de Linera-Alperi, Pablo Menéndez Fernández-Miranda, Bárbara Molina, Angel Batuecas-Caletrío, Nicolás Pérez-Fernández

**Affiliations:** 1 Department of Otorhinolaryngology, Clinica Universidad de Navarra, 28047 Madrid, Spain; mblancop@unav.es (M.B.); bmolinagil@unav.es (B.M.); nperezfer@unav.es (N.P.-F.); 2 Otoneurology Unit, Department of Otorhinolaryngology, Complejo Asistencial Universitario de Salamanca, IBSAL, University of Salamanca, 37008 Salamanca, Spain; chiaramonopoli@outlook.com (C.M.-R.); abatuc@yahoo.es (A.B.-C.); 3 Department of Radiology, Hospital Universitario Rey Juan Carlos, 28933 Madrid, Spain; pablo.mfernandez@quironsalud.es

**Keywords:** skull-vibration-induced nystagmus, visual fixation, peripheral vestibulopathy

## Abstract

Nystagmus induced by applying an intense vibratory stimulus to the skull (SVIN) indicates vestibular functional asymmetry. In unilateral vestibular loss, a 100 Hz bone-conducted vibration given to either mastoid immediately causes a primarily horizontal nystagmus. The test is performed in darkness to avoid visual fixation (VF) but there are no data about how much VF affects the often-intense SVIN. The aim is to analyze the amount of reduction in SVIN when VF is allowed during testing. Thus, all patients seen in a tertiary hospital for vertigo or dizziness with positive SVIN were included. SVIN was recorded for 10 s for each condition: without VF (aSVINwo) and with VF (aSVINw). We obtained an aSVINwo and an aSVINw as average slow-phase velocities (SPV) without and with VF. VF index (FI_SVIN_) was calculated as the ratio of SPV. Among the 124 patients included, spontaneous nystagmus (SN) was found in 25% and the median slow phase velocity (mSPV) (without VF) of SN was 2.6 ± 2.4°/s. Mean FI_SVIN_ was 0.27 ± 0.29. FI_SVIN_ was 0 in 42 patients, and FI_SVIN_ between 0 and 1 was found in 82 (mean FI_SVIN_ 0.39 ± 0.02). Fixation suppression was found in all patients with SVIN in cases of peripheral vestibulopathy. FI_SVIN_ clearly delineates two populations of patients: with or without a complete visual reduction in nystagmus.

## 1. Introduction

In 1973, Lücke described for the first time how a vibratory stimulus of 100 Hz applied by a vibrator to the mastoid process may provoke pathological nystagmus [1]. In later years, several authors further elaborated on the significance of this finding, as well as its relationship with the different pathologies affecting the vestibular system [2]. Thus, nowadays, it is well known that nystagmus induced by applying an intense vibratory stimulus to the skull (SVIN), mainly at the mastoid [3], indicates the existence of a functional asymmetry between the right and left side vestibular nuclei, mostly beginning at the level of the labyrinth [4]. It is therefore considered a test to perform at the bedside of patients seen for vertigo, dizziness and other vestibular symptoms. In patients with a unilateral vestibular loss (UVL), a 100 Hz bone-conducted vibration given to either mastoid triggers a primarily horizontal nystagmus with fast phases beating away from the affected side. In healthy, asymptomatic people, identical stimulation has little or no impact, since in the healthy subject the response to vibration would occur bilaterally and simultaneously, causing a nystagmus of the same speed, but in the opposite direction, and therefore would cause an annulment of this nystagmus [2,5]. In those few cases where healthy subjects develop nystagmus upon vibratory stimulation, the nystagmus response tends to be slow and inconsistent [6]. Moreover, SVIN has also been shown to be useful as a predictor of the follow up in patents with recurring vertigo and migraine symptoms. Among these patients, those who show predominantly SVIN are more likely to develop Menière’s disease than those who do not, while those who show positional nystagmus tend to develop Vestibular Migraine more frequently [7]. In this way, and as previously mentioned, this examination is considered a test to perform at bedside in routine clinical practice, since the SVIN can help not only to recognize the existence of an asymmetry in the functionality of both vestibular systems, but also in more specific aspects, such as the location of the injured side, in the case of vestibular neuritis, or in the diagnostic suspicion of Meniere’s disease in those patients with SVIN and recurrent vertigo [7,8].

Moreover, this type of nystagmus has some interesting features. There is no latency: in patients without spontaneous nystagmus (SN), the SVIN appears soon when the stimulus is applied to the skull, and when there is SN, its intensity increases or the direction also changes immediately. The intensity of the evoked nystagmus remains similar throughout the period of stimulation without its intensity or frequency reducing. It also disappears quickly when the stimulation ceases and surprisingly, whatever the intensity of the response, there is no reversion of nystagmus [9]. Some of these features would probably change given a prolonged period of stimulation; however, skull vibration is a bothersome type of mechanical testing that has some potential undesirable side effects, and it is not recommended to maintain that stimulus for more than 20–30 s. Some of the characteristics—absence of latency, habituation and slow adaptation and the marked coupling to the stimulus—are very reminiscent of other types of nystagmus, such as magnetic field-induced nystagmus [10].

In common practice this test is performed in complete darkness to avoid visual fixation [11] but, there are no data about how much visual fixation affects the often-intense SVIN. The visual fixation suppression of nystagmus is a common phenomenon that points to a peripheral origin of nystagmus, but it can also be seen in patients with central lesion, as in stroke [12].

A video head impulse test (vHIT) provides fast and accurate information on the functional status of the semicircular canal afferents in the high-frequency domain. Although it is considered that vHIT examines high-frequency sensible hair cells, while SVIN, as well as the caloric test, examine the low-frequency sensible vestibular hair cells [13], it has been shown that SVIN intensity correlates with the amount of vestibular deficit obtained in the vHIT [14,15]. A similar correlation has also been found in case of functional asymmetry between the two horizonal semicircular canals at the caloric test [16].

The aim of this study is to analyze the amount of SVIN reduction when visual fixation is allowed during the test, establishing as a main hypothesis that this reduction should be complete or almost complete. Moreover, given that in unilateral vestibulopathy the intensity of the SVIN depends on the severity of the vestibular damage, we would also try to make a first approach to analyze the relation between the amount of visual fixation effect and the gain of the vestibulo-ocular reflex (VOR).

## 2. Materials and Methods

### 2.1. Patients

We have included all patients seen in a tertiary hospital for vertigo or dizziness who underwent a complete oto-neurological exam and who during testing showed a positive SVIN. The SVIN examination was performed as part of the regular clinical evaluation of patients with vestibular symptoms.

All patients had normal eye position in primary gaze, and during the cover and cover–uncover test there was no large shift of both eyes; the visual acuity was sufficient to be able to see the red light while testing the effect of visual fixation [17].

### 2.2. Nystagmus Evaluation

In all subjects, the same video equipment was used for nystagmus registration and analysis: VisualEyes^®^525 by Interacosutics A/S (Middelfart, Denmark). 

Spontaneous nystagmus (SN): This was registered with the patient seated with gaze ahead, rightward and leftward, and in all of these with and without visual fixation. The time for registration was 30 s. SN was characterized by its direction and slow-phase velocity (SPV). All patients showed a complete or marked reduction in SN when visual fixation was allowed.

SVIN: We followed a previously used protocol. The SVIN was evoked in a sitting patient by stimulating both mastoid processes for 15 s using a 100 Hz handheld vibrator (VVIB 100; Synapsys, Marseille, France). Nystagmus evoked upon stimulation was recorded using videonystagmoscopy in the dark. Subjects were instructed to continue looking straight ahead during stimulation. SVIN was considered positive when the same nystagmus was recorded when the vibrator was placed on both the right and left mastoid: no directional dissociation. The nystagmus had to be present throughout the stimulation period, with an abrupt onset and an abrupt end, both coupled to the application of the vibrator. Once it was confirmed that directional dissociation was not occurring, the side where the intensity of nystagmus was greatest (measured by the maximum SPV) was the same side on which the SVIN response to visual fixation was verified. Then, SVIN was registered for 10 s without visual fixation and 10 s with visual fixation; for this, a red dot was presented in front of the left eye, which was the eye in which nystagmus was measured. Thus, we obtained an aSVINwo (in darkness) and an aSVINw (with visual fixation) as the average SPV without and with visual fixation. Only patients with an aSVINwo > 2°/s were considered for the study.

The visual fixation index for SVIN (FIsvin) was calculated as the ratio of slow phase velocities:

$$FIsvin=\frac{aSVINw}{aSVINwo}\times 100$$

In this formula, 0 result indicates a complete reduction or completely absent nystagmus during visual fixation; a higher score indicates that visual suppression is worse or incomplete.

### 2.3. Video Head-Impulse Test (vHIT)

This was carried out using a vHIT system that allows all 6 semicircular canals to be tested (GN Otometrics, Taastrup, Denmark). For this test, the patient wears a pair of lightweight, tightly fitting goggles on which is mounted a small video camera and a mirror that reflects the image of the patient’s right eye into the camera. The eye is illuminated by a low-level infra-red light-emitting diode. A small sensor on the goggles measures the head movement. Calibration is performed and the procedure of vestibulo-ocular testing is initiated. Horizontal semicircular canals are then tested: the clinician asks the patient to keep staring at an earth-fixed target 1 m in front, and gives the patient brief, abrupt head rotations through a small angle (about 10–20 degrees), unpredictably turning to the left or right on each trial. At the end of each head turn, the head-velocity stimulus and eye-velocity response, according to the stimulation of the horizontal semicircular canals, are displayed simultaneously on the screen, together with a graph of the calculated VOR gain (ratio of eye velocity to head velocity) for every head rotation. In a full test, 20 impulses are delivered randomly in each direction. Gain was evaluated as normal or abnormal according to norms by age [18]. A relative parameter was obtained to measure the gain asymmetry (Gass) between both sides:

$$Gass=\frac{Gh-Gl}{Gh+Gl}$$

In this formula, Gh was the highest gain value and Gl the lowest gain value, and in the case of patients with unilateral vestibulopathy (see later) they were also the unaffected and affected sides, respectively. The result extends from 0 when both gains were similar, and to 1 when one of them is 0.

### 2.4. Statistics

Quantitative variables were described using the mean and standard deviation, and the range. Qualitative variables were described using the percentage. Hypothesis contrasts were performed between FI_SVIN_ groups and the study variables. The chi-squared test was chosen when the study variables were categorical. In case of quantitative variables, the U-Mann–Whitney test was used, since the distributions were not normally distributed. Finally, Pearson’s correlation coefficient was used to study the correlation between the SVIN and VOR. A *p*-value of <0.05 was considered statistically significant.

**Figure 1:**
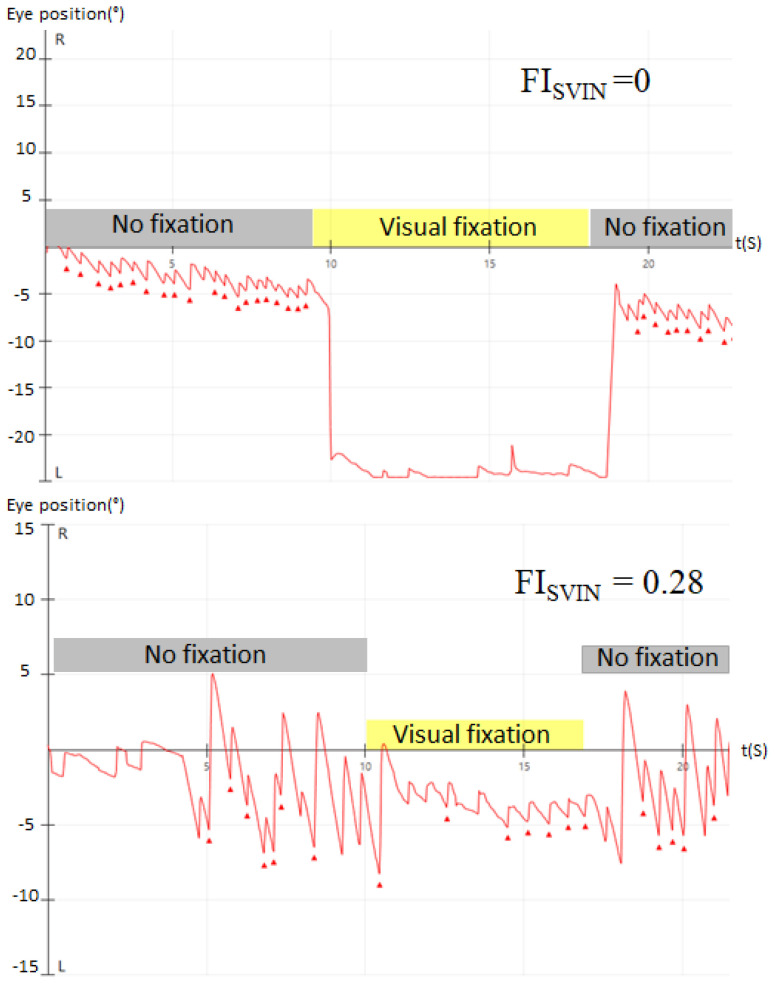
In this figure we present the results of two different patients. The upper graph shows a complete visual fixation effect on SVIN (FI_SVIN_ = 0), while the lower graph corresponds to a patient with incomplete visual fixation effect on SVIN (FI_SVIN_ = 0.28).

According to the effect of visual fixation on SVIN, the patients were classified into 2 groups: (1) complete visual fixation when the FI_SVIN_ = 0, and (2) incomplete visual fixation when >0 FI_SVIN_ <1 (Figure 1).

## 3. Results

We included 124 patients who, during the period of study, showed SVIN at their corresponding vestibular examination. There were 65 (52.5%) females and 59 (47.5%) males. The main diagnostic categories among patients were the following: 51 cases of Ménière’s disease (26 not previously treated, and 25 treated with intratympanic gentamicin), 26 sub-acute vestibular neuritis, 17 Benign Paroxysmal Positional Vertigo (BPPV), 16 recurrent non-positional vertigo, and 14 surgically treated vestibular schwannoma. The right side was affected in 59 (47.58%) patients and the left in 46 (37.10%); no side data were evident in 19 (15.32%) (Table 1). The 65 patients in whom there was a unilateral vestibulopathy (UVL) due to post gentamicin treatment, vestibular neuritis or post-surgical vestibular schwannomas will be the subject of a first approach to analyze the relation between the amount of visual fixation effect and the gain of the vestibulo-ocular reflex (VOR). 

In the complete group of patients, SN was found in 44 (35.5%) (Table 1) and the mSPV (without visual fixation) of SN was 2.6 ± 2.4°/s. The aSPV of SVIN (aSVINwo) was 11.2 ± 8.3°/s and when performed allowing visual fixation, the aSPVw of SVIN (aSVINw) was 3.6 ± 4.5°/s. The mean FI_SVIN_ was 0.27 ± 0.29. The FI_SVIN_ was 0 in 42 (33.8%) patients and >0 FI_SVIN_ <1 in 82 (66.2%) patients; in this group, the mean FI_SVIN_ was 0.37 ± 0.02. There were no significant differences for age (*p* = 0.590), sex (*p* = 0.269), ear affected (*p* = 0.135), SN (*p* = 1) or SN direction (*p* = 0.414) according to amount of FI_SVIN_. In Table 2, we present the data of main diagnoses and gain of the VOR asymmetry.

**Figure 2:**
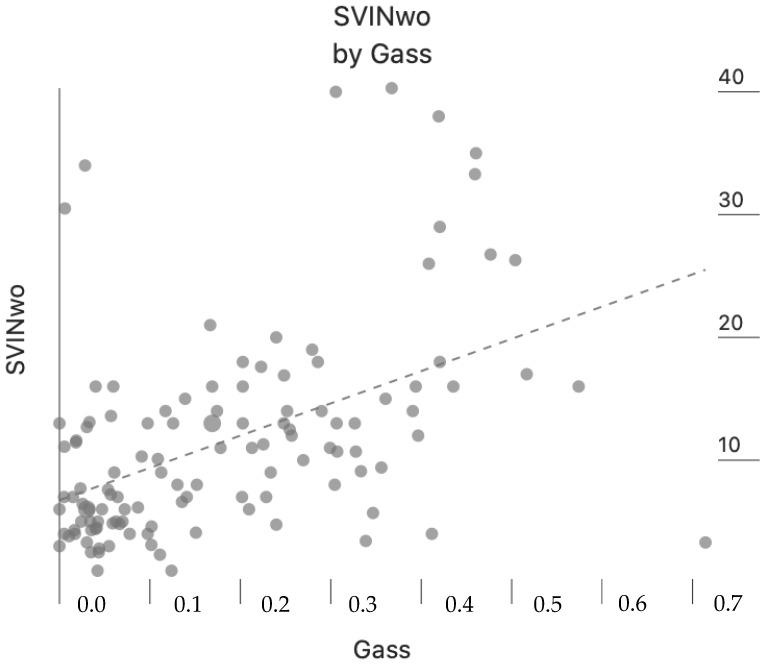
Correlation between the intensity of SVIN without visual fixation (SVINwo) and the degree of asymmetry of the VOR (Gass) in all the patients of the study.

In patients with no significant UVL, the gain of the VOR for head impulses towards the lowermost side was 0.86 ± 0.23 and to the opposite 0.99 ± 0.02. In the group of patients with UVL, the gain of the VOR for head impulses towards the affected side was 0.52 ± 0.02 and towards the unaffected 0.87 ± 0.16. Differences were significant for the mean Gass, aSVINwo and aSVINw between patients in the group without unilateral vestibulopathy and with unilateral vestibulopathy (*p* < 0.001 in the three cases). However, the amount of visual fixation was similar in both groups. In Figure 2, we present the intensity of SVINwo according to the gain asymmetry of the VOR for all the patients in the study (Pearson-Moment correlation, r = 0.484, *p* < 0.001).

**Figure 3:**
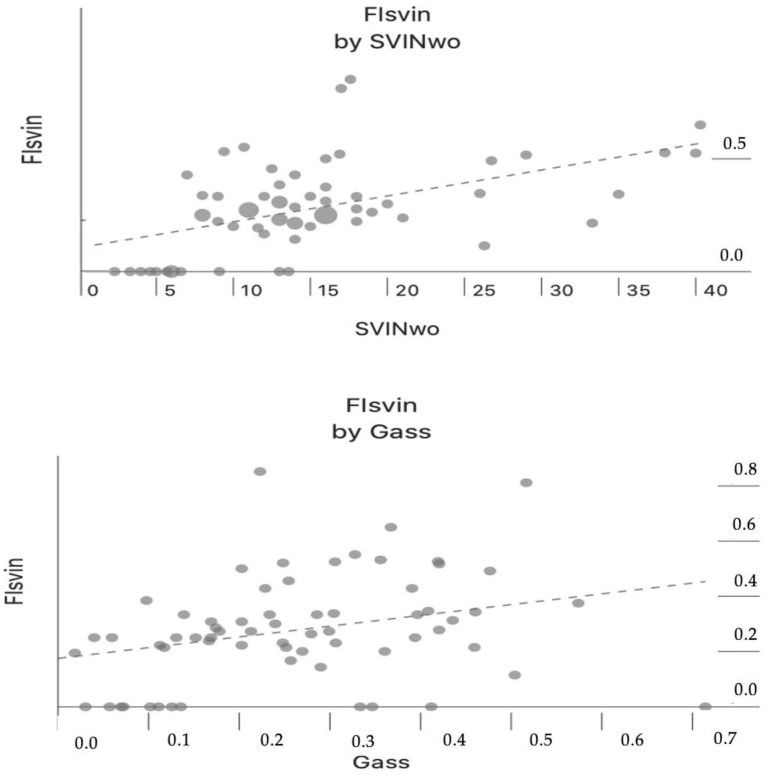
Patients with unilateral vestibulopathy: (**upper**) fixation index of SVIN (FIsvin) according to the intensity of SVIN without visual fixation; (**lower**) fixation index of SVIN (FIsvin) according to asymmetry of the VOR in the vHIT.

**Figure 4:**
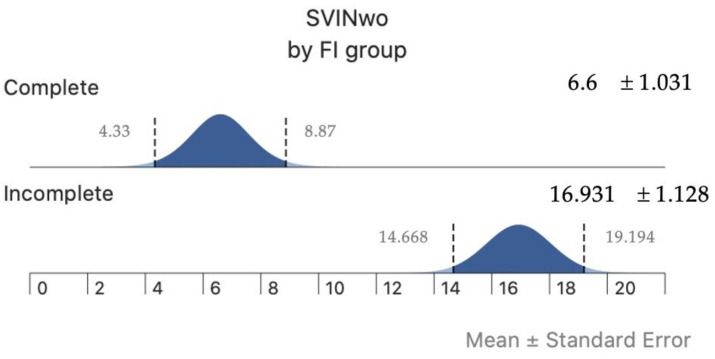
Intensity of SVIN (x-axis) according to the amount of reduction after visual fixation (complete vs. incomplete) in patients with unilateral vestibulopathy.

There was a positive correlation between aSVINwo and the FI_SVIN_ (Pearson correlation, r = 0.51, *p* < 0.001) only in patients with UVL; this indicates that when the intensity of SVIN is high, the amount of reduction by vision is lower (Figure 3upper). Both parameters (aSVINwo and FI_SVIN_) also showed a positive correlation with Gass (Pearson correlation, r = 0.39, *p* = 0.002 and Pearson correlation, r = 0.33, *p* < 0.001, respectively) only in patients with UVL (Figure 3lower and Figure 4, respectively). In the group of patients with UVL, the median Gass in patients with a complete visual suppression of SVIN was 0.118 and when incomplete it was 0.257, and differences were significantly different (U Mann–Whitney test, *p* = 0.041).

## 4. Discussion

In this work, we have shown that SVIN is suppressed by visual fixation in peripheral disorders. The amount of fixation can either be complete (no nystagmus during testing when allowing visual fixation) or incomplete (there is still some nystagmus during the visual task). All patients showed a reduction in the intensity of nystagmus, with incomplete fixation the most frequent finding. It has previously been reported that the intensity of SVIN (as measured by the aSVINwo) correlates to the degree of asymmetry in the VOR between the right and left vestibules [4,14]. In patients with UVL, the amount of reduction in SVIN as measured with the FI_SVIN_ correlates to both the asymmetry of gain of the VOR and the intensity of SVIN. When the asymmetry of VOR and/or the intensity of nystagmus are higher, then the degree of velocity reduction by visual fixation is lower. 

SVIN is a type of triggered nystagmus, but in this case it is evoked when applying a precise stimulus at both mastoids. It is expected to occur when there is a functional vestibular asymmetry between both sides. In our procedure (100 Hz stimulation), both the otoliths organs and semicircular canals are activated [19]. This probably explains SVIN in some cases in which the right–left VOR asymmetry is very low, such as patients with Ménière’s disease or recurrent non-positional vertigo, or even in cases without a specific vestibulopathy, such as patients with BPPV. Although the functional status of the saccule and utricle could also be the source of vestibular asymmetry and SVIN [20], unfortunately, we do not have this information in all patients in our study. However, the amount of reduction in nystagmus when visual fixation is allowed was the same in this group of patients as in those with UVL. The explanation of this finding could be related to a central effect [21], common to other spontaneous or induced nystagmus with [22] or without [23] permanent vestibular damage.

In addition, one of the main interests of the effect of visual fixation on SVIN lies in the fact that impaired fixation nystagmus suppression is frequently observed in central lesions and should be part of any nystagmus test evaluation [24]. This is an effect mediated by different parts of the cerebellum, but mainly in the flocculus, paraflocculus, nodule and uvula, so that when nystagmus is not suppressed by visual fixation, or even increases/appears, some degree of damage at a central level is suspected [25]. In these cases, there will also be additional signs of oculomotor dysfunction, as VOR visual suppression is mediated by smooth-pursuit cancellation.

Our results showed that visual suppression was complete in some cases. However, some other patients still showed nystagmus even when vision was allowed. When the whole population is considered, the amount of visual fixation is very much like that shown in caloric nystagmus and per-rotatory nystagmus in patients with peripheral vestibulopathy and also with the later test in controls: the mean reduction found by authors was 80% [26]. However, when patients with some response during visual fixation are considered, then the mean fixation index falls to 0.4. It will be interesting to analyze whether, with time and in the case of vestibular periphery functional recovery, as occurs in some cases after intratympanic gentamicin [27], there is some modification of the fixation index.

We have not included patients with central vestibulopathy because of the unusual finding of SVIN [28] and because nystagmus is often vertical [29]; in this sense, the peripheral counterpart in which vertical SVIN is more common are those patients with superior semicircular dehiscence. In those cases, stimulation should preferentially be performed at the vertex and the most suitable frequency (500 Hz) is above the one used in our work (100 Hz) [30].

The second interest in visual fixation in SVIN rests in the effects of skull vibration in the light or visual fixation condition. It has been reported that both the horizontal and vertical components of nystagmus in patients with non-acute unilateral vestibular loss have been abolished (as measured in our work), but there is a torsional shift of the eye of approximately 3°–6° towards the affected side, no matter the mastoid side stimulated [31]. The degree of torsion is only related to the amount of vestibular damage when the vibration is on the sternocleidomastoid muscles but not on the mastoid. This effect is also seen in normal subjects and explains the modification of visual orientation perception in the roll plane [32]. We have not evaluated this finding, which should also be assessed with the corresponding fixation index value and otolithic functional testing.

In conclusion, since fixation suppression was found in all patients with SVIN in cases of peripheral vestibulopathy, it is of interest as a confirmatory bedside test. When quantified by any of the different nystagmography methods, the fixation index clearly delineates two populations of patients: with or without a complete visual reduction in nystagmus. Better measures of vestibular function that implicate visual–vestibular interaction need to be considered.

## Figures and Tables

**Table 1:** Descriptive statistics of qualitative variables.

Variable	n	(%)
Sex		
Female	65	52.5
Male	59	47.5
Diagnosis		
Ménière’s disease	51	41.1
Sub-acute vestibular neuritis	26	21.0
BPPV	17	13.7
Recurrent non-positional vertigo	16	12.9
Post-surgical vestibular schwannoma	14	11.3
Affected side		
Right	59	47.6
Left	46	37.1
NA	19	15.3
Spontaneous nystagmus		
Yes	44	35.5
No	80	64.5

BPPV = Benign Paroxysmal Positional Vertigo; NA = missing values.

**Table 2:** Mean ± standard deviation of quantitative variables for each patient group.

Diagnosis	Gasym	aSVINwo	aSVINw	FI_SVIN_
Ménière’s disease				
Untreated	0.1 ± 0.1	9.7 ± 6	3 ± 3.6	0.29 ± 0.2
Post-gentamycin	0.13 ± 0.9	12 ± 7	3.2 ± 4	0.21 ± 0.14
Sub-acute vestibular neuritis	0.36 ± 0.15	15 ± 9	5.3 ± 6	0.3 ± 0.2
Post-surgical vestibular schwannoma	0.28 ± 0.14	16 ± 9.5	6 ± 5.5	0.3 ± 0.17
Recurrent vertigo	0.08 ± 0.09	5.3 ± 3.5	2 ± 2.7	0.29 ± 0.49
Positional (BPPV)	0.03 ± 0.02	7.8 ± 8	2.2 ± 3.3	0.25 ± 0.37
Non-positional	0.03 ± 0.02	7.8 ± 8	2.2 ± 3.3	0.25 ± 0.37

Gasym = Gain of VOR asymmetry; aSVINwo and aSVINw = average slow phase of nystagmus velocity of SVIN without (wo) and with (w) visual fixation; FI_SVIN_ = fixation index of SVIN by diagnoses.

## Data Availability

The data presented in this study are available upon request to the corresponding author because they are patient data that constitute sensitive information.
